# Correlates and Determinants of Cardiorespiratory Fitness in Adults: a Systematic Review

**DOI:** 10.1186/s40798-019-0211-2

**Published:** 2019-09-03

**Authors:** Johannes Zeiher, Katherine J. Ombrellaro, Nita Perumal, Thomas Keil, Gert B. M. Mensink, Jonas D. Finger

**Affiliations:** 1 Department of Epidemiology and Health Monitoring, Robert Koch Institute, General-Pape-Straße 62-66, 12101 Berlin, Germany; 2 Department of Infectious Disease Epidemiology, Robert Koch Institute, Berlin, Germany; 3 Institute for Clinical Epidemiology and Biometry, University of Würzburg, Würzburg, Germany; 4 Institute for Health Resort Medicine and Health Promotion, Bavarian Health and Food Safety Authority, Bad Kissingen, Germany; 5 Institute for Social Medicine, Epidemiology and Health Economics, Charité - Universitätsmedizin Berlin, Berlin, Germany

**Keywords:** Cardiorespiratory fitness, Aerobic fitness, Risk factors, Individual factors, VO_2max_, Health behavior, Systematic review

## Abstract

**Background:**

Enhanced cardiorespiratory fitness (CRF) is now a well-established predictor of numerous adverse health outcomes. Knowledge about the pathways leading to enhanced CRF is essential for developing appropriate interventions. Hence, the aim of this review was to provide a detailed overview of the current state of research regarding individual factors associated with or influencing CRF among the general adult population.

**Methods:**

We searched the PubMed, EMBASE, and Cochrane Library databases and also conducted a search for grey literature (Google Scholar). Eligible indicators of CRF were objectively assessed measures of CRF by submaximal or maximal exercise testing measured using treadmill or cycle ergometer tests. We included quantitative observational studies of the general adult population. Using a semi-quantitative approach, we compiled summary tables aggregating the study results for each potential correlate or determinant of CRF.

**Results:**

We identified 3005 studies, 78 of which met the inclusion criteria. Almost all of these studies were conducted in high-income countries. Study quality scores assessing the risk of bias in the individual studies ranged from 40 to 100%. Male sex, age (inverse), education, socioeconomic status, ethnicity, body mass index (inverse), body weight (inverse), waist circumference, body fat (inverse), resting heart rate (inverse), C-reactive protein (inverse), smoking (inverse), alcohol consumption, and multiple measures of leisure-time physical activity were independently and consistently associated with CRF.

**Conclusions:**

In synthesizing the current research on the correlates and determinants of CRF among adults, this systematic review identified gaps in the current understanding of factors influencing CRF. Beyond the scope of this review, environmental and interpersonal determinants should be further investigated.

**Systematic Review Registration:**

PROSPERO, CRD42017055456.

**Electronic supplementary material:**

The online version of this article (10.1186/s40798-019-0211-2) contains supplementary material, which is available to authorized users.

## Key Points

- This study is a systematic review of evidence concerning the correlates and determinants of CRF among adults in 78 included studies, which were conducted in 20 countries.
- Whereas factors such as age and waist circumference were consistently associated with cardiorespiratory fitness, there was conflicting evidence for many other factors, revealing research gaps for future studies to address.
- This comprehensive summary of a large body of evidence may be used to develop evidence-based interventions to improve fitness levels in the general adult population.

## Background

A key aspect of the global strategy to tackle non-communicable diseases (NCDs) is the promotion of physical activity (PA) and the reduction of sedentary behavior [79, 80]. PA has been linked to positive health outcomes, such as lower risks of ischemic heart disease, stroke, diabetes, and depression, and to a reduction in all-cause mortality [81]. Cardiorespiratory fitness (CRF) is another dimension of physical health linked to beneficial health outcomes. Whereas PA is behavioral and can be described as any bodily movement that is produced by skeletal muscles and requires energy exposure, CRF is a trait and is defined as the ability of the circulatory, respiratory, and muscular systems to supply oxygen during prolonged moderate-to-vigorous dynamic exercise [82, 83]. Therefore, PA and CRF are related, but not identical. CRF is usually measured using treadmill or ergometer exercise tests and is often expressed as maximal oxygen consumption (VO_2max_), whereas PA is often assessed through self-report.

Over the past 30 years, research has shown that the positive effects of enhanced physical fitness, and especially CRF, are comparable to or even greater than those of PA [85–89]. In addition to predicting all-cause mortality, low CRF is an established predictor of cancer mortality [90], depression [91, 92], and metabolic syndrome [93, 94]. Among the risk factors for cardiovascular disease (CVD), poor CRF has been found to be the most powerful predictor of morbidity [95].

Because of the importance of CRF in NCD prevention, it is crucial to better understand the correlates and determinants of CRF in the general population. CRF is known to be partly genetically determined [96, 97]. In addition to hereditary determinants and PA, many other individual and environmental factors are presumed to influence CRF [84, 88, 98]. A growing body of work links CRF to factors such as age [99], sex [100], smoking [8], alcohol consumption [101], body mass index (BMI) [23], educational status [102], and the residential built environment [103]. Although initial attempts have been made to develop a model of CRF and its determinants [84, 98], so far, there is no comprehensive model or framework that incorporates a wide range of influencing factors and the interrelations among them, as has been done in models of obesity [104, 105] and PA [106, 107]. Moreover, although researchers have systematically reviewed the factors associated with PA [106], to our knowledge, there has been no systematic review of the factors associated with CRF. Knowledge about the various pathways leading to the development of fitness is essential for creating appropriate interventions.

Hence, the aim of this systematic review was to provide a detailed overview of the current state of research regarding factors associated with (“correlates”) or influencing (“determinants”) CRF among the general adult population. Furthermore, we aimed to analyze the consistency of the reported associations. To narrow the study focus, we concentrated on individual factors associated with CRF, omitting interpersonal and environmental correlates and determinants.

## Methods

### Protocol and Registration

The review methodology, including the search strategy, data collection, and quality assessment of the included studies, was pre-specified and has been published in a review protocol [108]. The review was registered in the International Prospective Register of Systematic Reviews (PROSPERO, CRD42017055456). We followed the Preferred Reporting Items for Systematic Reviews and Meta-analyses (PRISMA) guidelines whenever applicable.

### Search Strategy and Eligibility Criteria

Relevant studies were located from different sources: We searched the PubMed, EMBASE, and Cochrane Library databases from inception to Present. The last search was run on 1 February 2017. In addition, we conducted a search for grey literature (Google Scholar). A limited update literature search was performed from 01 February 2017 to 6 June 2019 on PubMed. We used a broad range of search terms for CRF measures (outcome; e.g., “cardiorespiratory fitness”) and general correlates and determinants (exposures; e.g., “health behavior”) to ensure that all potentially relevant articles investigating the factors associated with CRF were included [108]. We also cross-checked the references of the articles selected for full-text screening to locate additional studies. No language, text availability, publication status, or date restrictions were imposed. Eligible indicators of CRF were objectively assessed measures of CRF by submaximal or maximal exercise testing. Therefore, both direct indicators measured via spiroergometry and indirect indicators calculated via metabolic equations of oxygen consumption were included. We only included studies that assessed CRF by treadmill or cycle ergometer. The preferred laboratory measure of CRF (the “gold standard”) is maximal oxygen consumption (VO_2max_), which is measured in milliliters per kilogram per minute (ml/kg/min) during exercise and reflects a person’s maximal ability in terms of oxygen uptake, use, and transport [109]. VO_2max_ is defined as the point when oxygen consumption reaches a plateau and cannot be increased with an increase in effort [83, 110]. However, in exercise testing, such a clear plateau often cannot be achieved, and, instead, the highest obtained VO_2_ value, regardless the subject’s effort, (VO_2peak_) is used [110]. Because the distinction between these two measures was not always clear in the included studies [111], this review uses the term VO_2max_ for both VO_2max_ and VO_2peak_.

**Fig. 1:**
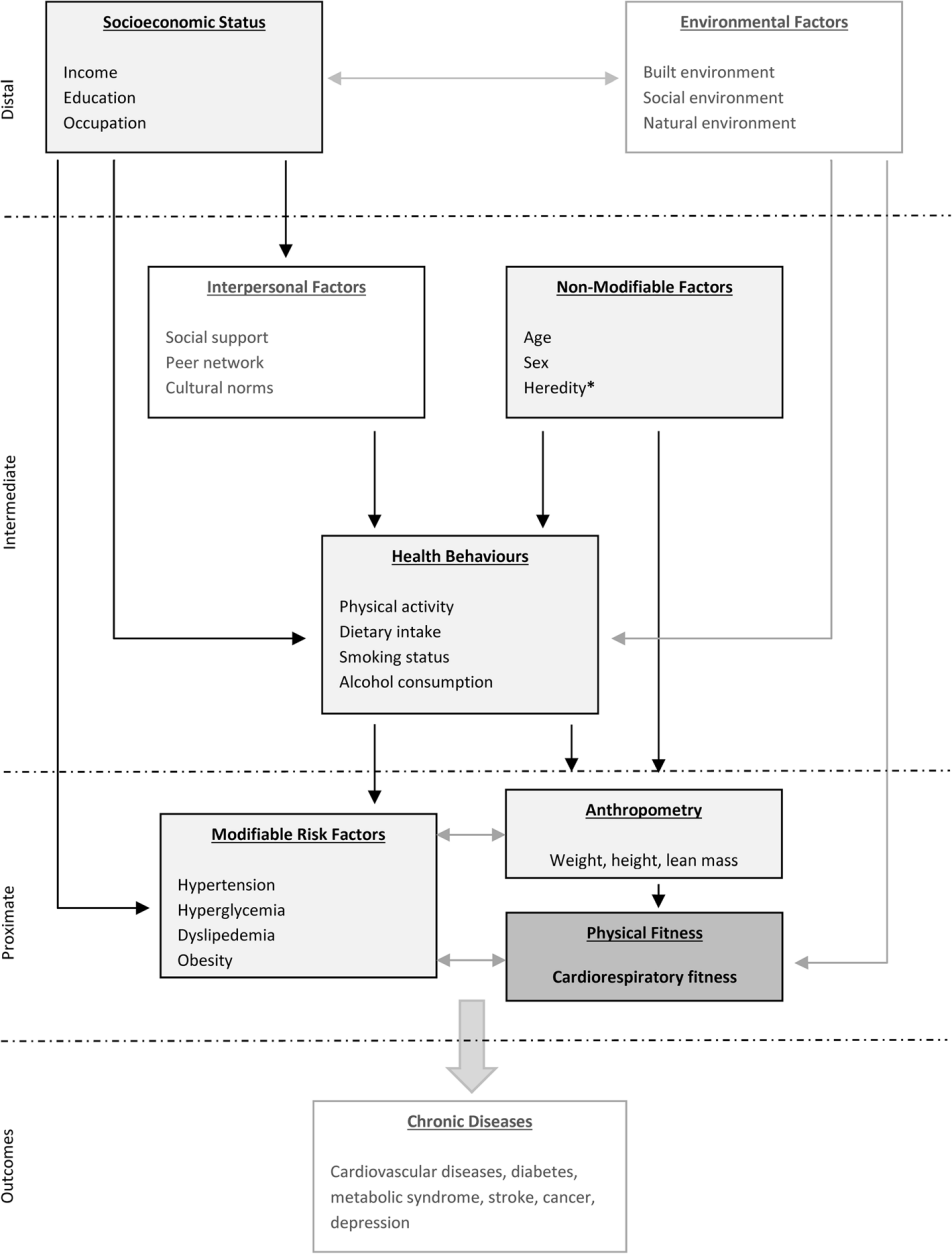
Conceptual framework of the correlates and determinants of cardiorespiratory fitness. Light grey boxes: potential individual correlates and determinants of cardiorespiratory fitness. *Genetic factors were not in the scope of this review

To structure our search strategy, we used a conceptual framework that locates CRF on the pathway to NCDs and defines and categorizes potential factors associated with CRF. Based on the method recommended by Victoria et al. [112], we proposed this framework by adopting elements of different ecological models [108] (Fig. 1).

As mentioned above, to limit the scope of this systematic review, we focused on individual correlates and determinants of CRF and excluded environmental (e.g., public green spaces) and interpersonal (e.g., social support) factors. The following categories of factors were considered: (1) sociodemographic factors (e.g., age, sex, and education); (2) anthropometric measures (e.g., BMI, weight, and waist circumference [WC]; (3) vital parameters (e.g., resting heart rate [HR] and blood pressure [BP]); (4) comorbidities and medications; (5) biomarkers (e.g., C-reactive protein [CRP]); (6) PA parameters (e.g., leisure-time PA [LTPA]); and (7) other health-related behaviors (e.g., smoking and nutrition). Genetic factors, such as specific genetic variants associated with CRF trainability [113], were not included in this review.

In this review, we included quantitative observational studies (cohort studies, case–control studies, and cross-sectional studies) reporting individual correlates of CRF in the general adult population. The following exclusion criteria were applied: (1) studies on interventions or trials promoting CRF or PA; (2) studies focusing on children (aged 0–11 years), adolescents (aged 12–17 years), or older adults (aged 80+ years), although studies investigating a broad age range were not excluded and may include participants aged younger than 18 years or older than 80 years; (3) studies with highly select groups of participants not representative of CRF/PA in the general population (e.g., specific occupations such as firefighters or special forces, athletes, sports students, or institutionalized populations such as patients in hospitals or nursing homes); (4) studies presenting only univariate results that were unadjusted for confounding; (5) studies that assessed CRF by means other than bicycle ergometer or treadmill; and (6) reviews, letters to the editor, commentaries, and editorials.

### Data Extraction and Quality Assessment

We imported all search results into Endnote X7 (Thomas Reuters, USA) reference management software and removed duplicates. At the first stage of screening, we reviewed the titles and abstracts. At the second stage, we assessed full-text articles for eligibility. The assessment was performed by two independent reviewers (NP and KJO at the first stage, JZ and KJO at the second stage). If the reviewers disagreed, a third reviewer was included in the discussion (JDF). Final decisions were made by consensus. From the final included studies, the following information was extracted and transferred to a detailed extraction worksheet, which was developed and pilot tested in advance: study characteristics (title, year, study name, country, and study period), study methods (e.g., study type, sample size, and inclusion/exclusion criteria), study population characteristics (e.g., age range and sex ratio), details of CRF assessment (e.g., test machine and exercise test protocol), analytical methods (e.g., statistical method and type of reported outcome), results, and reported limitations (Additional file 4: Data extraction table). When different multivariable models were reported for a sample, we extracted the fully adjusted models. The data extraction was conducted independently by two authors (JZ and KJO). Disagreements were solved by discussion; if agreement could not be reached, a third author (JDF) was involved, and decisions were made by a simple majority. The limited search update was performed by two authors (JZ and JDF [10% random sample at title and abstract screening, full sample at full text screening]). Information from eligible studies retrieved in the update was extracted to a separate table (Additional file 3: Table OR1).

Additional information was obtained through a request to the author for one study, where further results were mentioned but not presented in detail [49]. The author responded, but this additional information was not used because the results violated the inclusion criteria. We contacted authors of two studies to clarify the direction of a reported association [18] and for further details about the study period [65]. Both of these authors responded, and the additional information was included in this study.

Risk of bias in each study was also assessed independently by two authors (JZ and KJO) using a customized version of the Quality Assessment Tool for Observational Cohort and Cross-Sectional Studies, published by the National Heart, Lung, and Blood Institute at the National Institutes of Health, USA [114]. We categorized risk of bias as “high” when a study reached < 50% of the fulfillment score, “moderate” when a study reached 50–75%, and “low” when > 75% of the criteria were fulfilled. Further details about this risk assessment procedure have been published elsewhere [108].

### Coding and Summarizing

We compiled summary tables aggregating the existing research on each potential correlate or determinant of CRF. For this purpose, we modified the “semi-quantitative approach” originally proposed by Sallis et al. [115] (adapted by [116, 117]). This approach allowed summary measures to be calculated for each analyzed exposure, even when the heterogeneity among studies was high and no meta-analysis was possible. Following this approach, in the present article, we distinguish between the terms “study” and “sample.” Each article included in this review is referred to as a study. Studies where the results were presented separately for men and women were counted as two samples. Studies that presented results only for the total population (men and women) and those that focused on only men or women were counted as one sample. Hereafter, we use the term “study” to refer to each article included in this review and the term “sample” to refer to each (sex-specific) sample or subsample.

For each sample, a significant direct or inverse association between CRF and the exposure is presented as “+” or “−” in the column “related to CRF”; non-significant associations are shown in the column “unrelated to CRF” (“0”). The findings are summarized by presenting the total number of samples and the numbers of samples with direct, indirect, and non-significant associations. Finally, following an approach applied in other semi-quantitative reviews [115–117], a summary measure for each association was calculated as follows: Agreement in direction in at least 60% of all samples was graded as a positive (“+”), negative (“−”), or non-significant association (“0”). If none of the categories had a majority of at least 60%, the correlate was assessed as unclear (“?”). Outcomes that were heavily investigated (i.e., in at least ten independent samples) for which there was result agreement in at least 80% of the samples are denoted by “++,” “− −,” or “00.” Summary measures were calculated only if an exposure was investigated in three or more individual samples; otherwise, the correlate was marked as not applicable (“n/a”). Each unique association is reported separately. Separate studies drawing on the same study population and reporting redundant exposures are presented in parentheses and were counted as one unit of analysis. For example, the two studies using the National Health and Nutrition Examination Survey data from the same study period that presented data on the association of age with CRF are both listed in the summary table but were counted as only one sample. In rare cases when separate studies using the same sample had contradictory results, we present the relevant details in a footnote.

#### Outcome Variable: CRF

For each sample included in this review, we extracted only one CRF measure, even if multiple measures were reported. Where it was assessed, we used the results for VO_2max_ because this is the gold standard for measuring CRF. If VO_2max_ was not reported, we summarize the results for the reported measures if they showed the same direction. When the results varied regarding the reported association with a specific exposure, we report the divergent results in a note at the end of the summary table. Additional information is also provided as a footnote to this table for cases where the association between CRF and a given exposure was non-linear (e.g., U-shaped) or the association was reported only for subgroups divided by variables other than sex (e.g., by ethnicity).

#### Exposure Variables: Individual Correlates and Determinants of CRF

We extracted each individual correlate or determinant of CRF and grouped them into the following categories: sociodemographic factors, anthropometric measures, vital parameters, comorbidities and medications, biomarkers, activity parameters, and other health behaviors. Correlates or determinants considered as individual factors that were not assignable to one of these categories (e.g., preterm birth) were grouped under “other.” Where possible, we clustered similar factors to enable the calculation of summary measures.

We performed sensitivity analyses for all exposures that were reported in at least ten samples. We cross-tabulated the numbers of samples with positive associations, negative associations, and null findings by sex (men/women/mixed sample), test machine (cycle ergometer/treadmill), and CRF measure (VO_2max_, direct/VO_2max_, indirect/other) and checked for significant differences using Fisher’s exact test. Using the same approach, we checked whether the results for BMI varied across CRF measures adjusting for body weight vs. measures that did not adjust for body weight.

## Results

### Study Characteristics

**Fig. 2:**
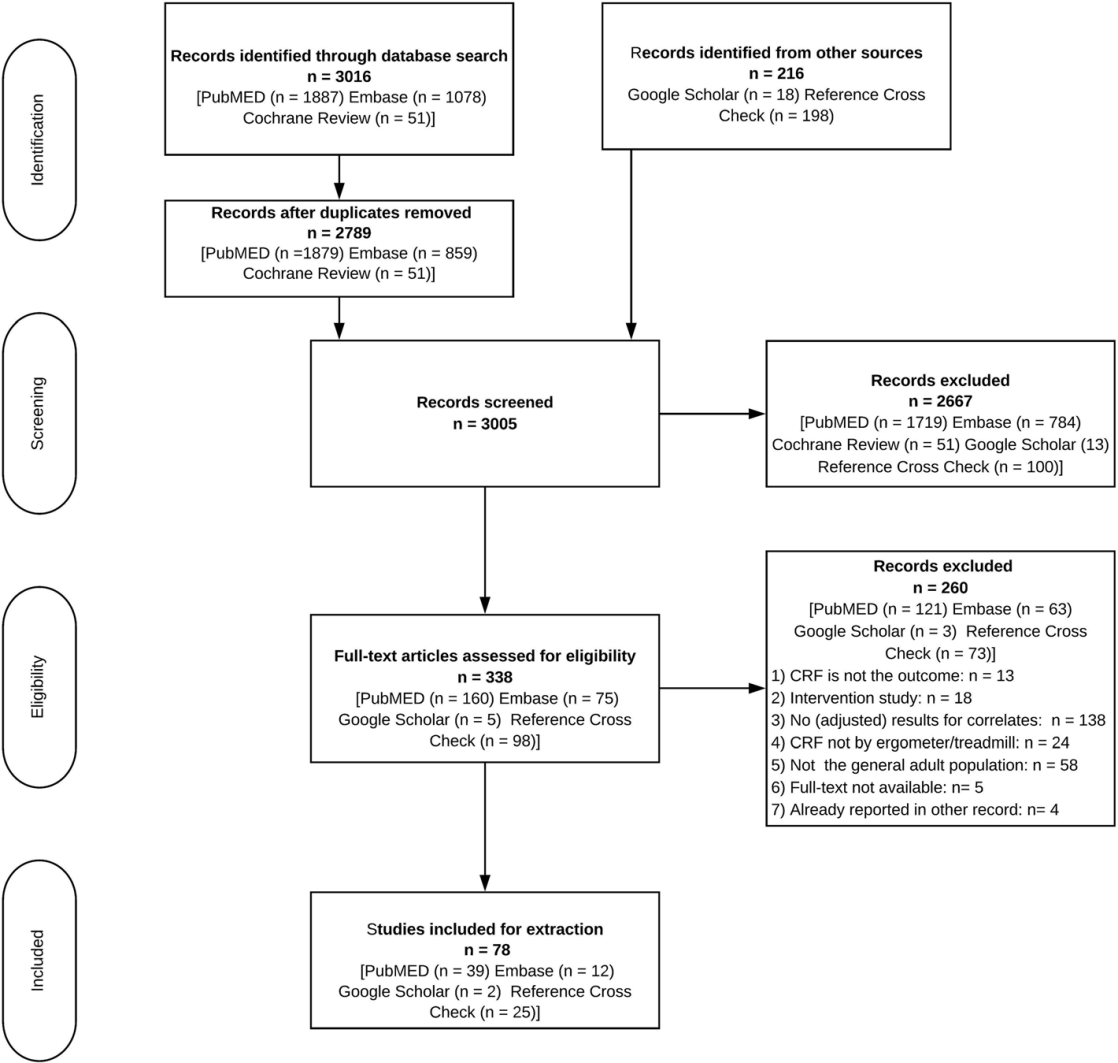
PRISMA flow diagram. PRISMA: Preferred Reporting Items for Systematic Reviews and Meta-Analyses

We identified a total of 78 articles for inclusion in this review. The complete selection process is shown in the PRISMA Flow Diagram (Fig. 2).

**Fig. 3:**
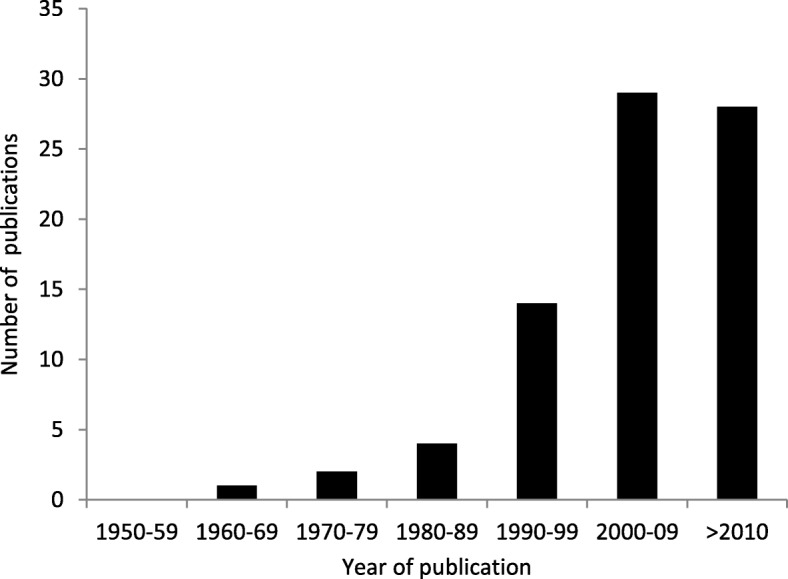
Distribution of publications by year of publication (*n* = 78)

The initial search in PubMed, EMBASE, and the Cochrane Library yielded 3016 records. Following the removal of duplicates, 216 articles were added from Google Scholar and the reference cross-check. After title and abstract screening, 338 records remained for full-text screening. A total of 260 of these records did not fulfil the eligibility criteria. All included studies were published in English. The dates of publication ranged from 1966 to 2017; the vast majority of articles were published after 2000 (see Fig. 3).

General characteristics of the studies reviewed can be found in Table 1.

**Table 1:** Included studies categorized by study characteristics

	Number of studies	%	Reference numbers
Risk of bias			
Low	30	38%	[1, 2, 4, 12, 14–16, 18, 20, 23–26, 31, 34, 36, 40, 42–44, 49, 50, 57, 59, 60, 62, 67, 75–77]
Medium	41	53%	[5–9, 13, 17, 21, 22, 28–30, 32, 33, 35, 37–39, 45–48, 51, 55, 56, 58, 61, 63–66, 68–74, 78]
High	7	9%	[3, 19, 27, 41, 52–54]
Sample size			
< 100	5	6%	[5, 17, 41, 53, 71]
100–299	19	24%	[4, 6, 7, 9, 10, 13, 21, 27, 29, 32, 48, 51, 52, 66, 68, 70, 73, 75, 76]
300–499	9	12%	[3, 8, 16, 33, 38, 39, 49, 72, 78]
500–1999	25	32%	[15, 18, 19, 22–26, 30, 31, 35, 40, 43, 44, 46, 47, 50, 54, 55, 60, 61, 63, 65, 69, 74]
2000–4999	14	18%	[1, 12, 14, 20, 34, 36, 42, 45, 56, 57, 59, 62, 67, 77]
> 5000	6	8%	[2, 11, 28, 37, 58, 64]
Region			
North America	31	40%	[1, 2, 4, 5, 10–12, 14, 17, 19, 21, 22, 28, 33, 34, 37, 42–44, 50–53, 57, 59, 60, 63, 65, 67, 69, 77]
Europe	29	37%	[6, 7, 18, 20, 23–27, 29, 30, 36, 38, 40, 45, 47–49, 55, 56, 62, 64, 66, 70, 71, 75, 76]
Asia	14	18%	[13, 16, 32, 35, 39, 41, 54, 58, 61, 68, 72–74, 78]
Oceania (including Australia)	4	5%	[3, 15, 31, 46]
World Bank income classification			
High-income countries	74	95%	[1–29, 33–60, 62–71, 74–78]
Upper-middle-income countries	2	3%	[72, 73]
Lower-middle-income countries	2	3%	[32, 61]
Low-income countries	0	0%	–
Country			
Canada	1	1%	[51]
United States	30	38%	[1, 2, 4, 5, 10–12, 14, 17, 19, 21, 22, 28, 33, 34, 37, 42–44, 50, 52, 53, 57, 59, 60, 63, 65, 67, 69, 77]
Finland	5	6%	[23, 30, 45, 47, 66]
Sweden	3	4%	[6, 7, 64]
Norway	3	4%	[18, 55, 62]
Netherlands	4	5%	[8, 38, 70, 71]
Germany	6	8%	[20, 25, 26, 29, 36, 40]
United Kingdom	2	3%	[24, 49]
Belgium	2	3%	[48, 76]
Lithuania	1	1%	[27]
Italy	1	1%	[56]
Spain	1	1%	[75]
Israel	3	4%	[16, 35, 58]
Jordan	1	1%	[32]
India	1	1%	[61]
China	2	3%	[72, 73]
Korea	2	3%	[39, 78]
Japan	5	6%	[13, 41, 54, 68, 74]
Australia	2	3%	[15]
New Zealand	2	3%	[31, 46]
Multiple countries	1	1%	[9]
Study design			
Cross-sectional	59	76%	[1–3, 9–14, 16, 18–21, 23–30, 32–36, 39–45, 47–52, 54, 56–59, 61, 62, 65–69, 72, 74, 75, 77, 78]
Longitudinal	18	23%	[6, 15, 17, 22, 31, 37, 38, 46, 53, 55, 60, 63, 64, 70, 73, 76]
Case–control	1	1%	[71]
Sex			
Women only	8	10%	[13, 19, 28, 39, 52, 53, 63, 73]
Men only	17	22%	[3, 5, 10, 17, 23, 32, 35, 45, 47, 48, 50, 55, 56, 61, 64, 74, 76]
Women and men	48	62%	[2, 4, 6–8, 11, 12, 14–16, 18, 20–22, 24–27, 29, 30, 33, 34, 36–38, 40–44, 49, 51, 54, 57–60, 65–72, 75, 78, 79]
NR	5	6%	[1, 9, 31, 46, 62]
Maximal or submaximal exercise testing			
Maximal	48	62%	[8, 11–13, 16–18, 21–23, 25–30, 33–40, 45, 47, 48, 51–53, 55–57, 59–61, 63–66, 69–71, 73–75, 77]
Submaximal	27	35%	[1, 5–7, 9, 14, 15, 19, 20, 24, 31, 41–44, 46, 49, 50, 54, 58, 62, 67, 68, 72, 76]
NR	3	4%	[10, 32, 78]
Exercise test machine			
Cycle	34	44%	[3, 5–7, 9, 13, 15, 20, 23–27, 30–32, 36, 40, 45–48, 51, 55, 56, 64, 66, 68, 71–76]
Treadmill	44	56%	[1, 2, 4, 8, 10–12, 14, 16–19, 21, 22, 28, 29, 33–35, 37–39, 41–44, 49, 50, 52–54, 57–63, 65, 67, 69, 70, 77, 78]
CRF measure			
VO_2max_, direct (among others)	39	50%	[4, 5, 8, 10, 13, 17, 18, 21–27, 29, 30, 33, 35, 38–40, 45, 47–49, 51–54, 62, 65, 66, 69–73, 75, 76]
VO_2max_ indirect (among others)	19	24%	[1, 6, 7, 9, 14, 16, 31, 41–44, 46, 50, 67, 68, 74, 77]
Only other measures	19	24%	[11, 12, 15, 19, 20, 28, 32, 34, 36, 37, 55–61, 63, 64]
NR	1	1%	[78]

*CRF* cardiorespiratory fitness, *NR* not reported, *VO*_2*max*_ maximal oxygen consumption

Most studies were from North America (31) or Europe (29), and there were 14 from Asia and four from Oceania (including Australia). Thus, almost all studies were from high-income countries (74), according to the World Bank classification; only four were from lower-middle or upper-middle-income countries, and none were from low-income countries. Except for one case–control study, all of the studies were cross-sectional (59) or cohort (18) studies. Almost two-thirds of the studies investigated both men and women (48), whereas eight studies reported results only for women and 17 reported results only for men. Five studies did not report the sex ratio of the study participants. The age range across the studies was 13 to 96 years. Sample sizes ranged from 79 to 218,820. A variety of statistical techniques were used to investigate the associations between CRF and potential correlates and determinants, including general linear models such as analysis of variance; analysis of covariance; (multiple) linear, logistic, and quadratic regression; partial correlation; and general estimating equations.

The quality scores assessing the risk of bias in the individual studies ranged from 40 to 100% (Additional file 1: Table OR1.1). Thirty studies were classified as having low risk of bias, 41 were classified as having medium risk of bias, and seven were classified as having high risk of bias.

### CRF

**Fig. 4:**
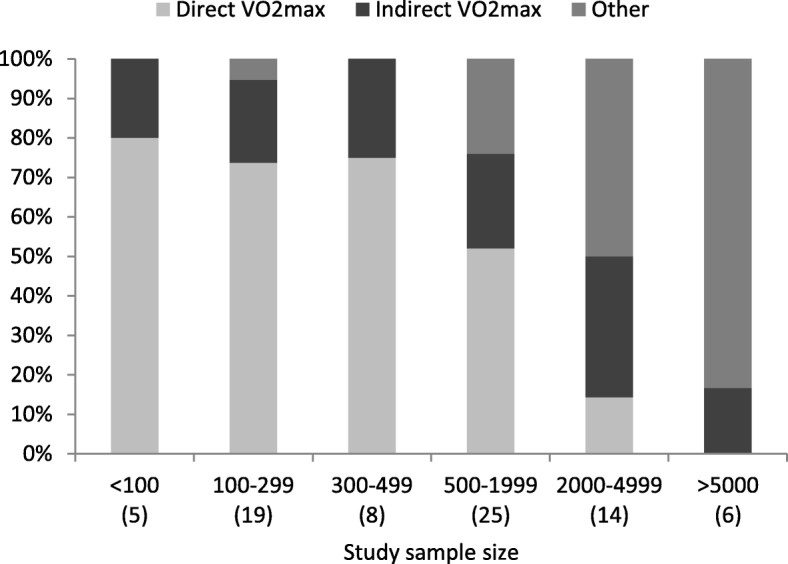
Cardiorespiratory fitness measurement used by study sample size (the number of studies appears in parentheses. The studies sum to *n* = 77 because one study did not report whether they used direct or indirect VO_2max_) *VO*_2*max*_ maximal oxygen consumption

Half of the studies (39) used directly assessed VO_2max_ via gas analysis as the outcome variable, 19 reported estimated VO_2max_, and 19 used only measures other than VO_2max_ as the outcome variables. Directly assessed VO_2max_ via gas analysis was more common in studies with smaller sample sizes. No studies with more than 5000 participants performed gas analysis to asses VO_2max_ (see Fig. 4).

**Fig. 5:**
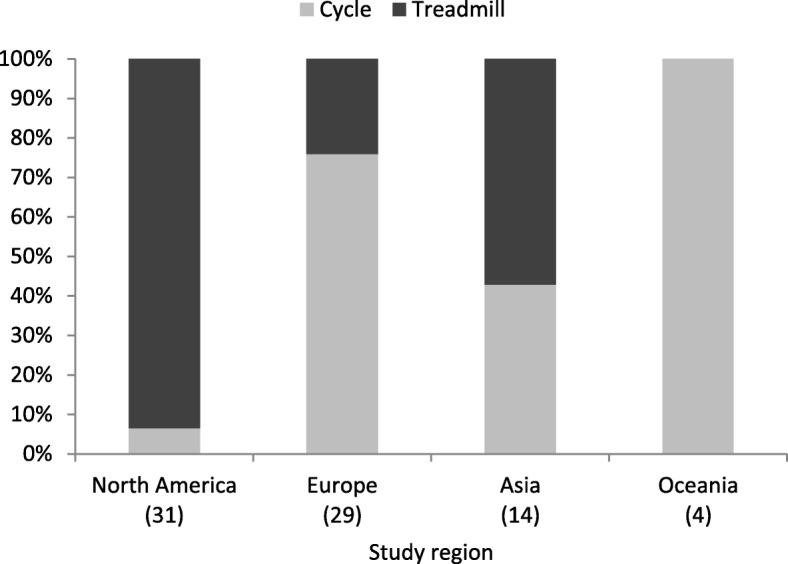
Exercise test machine used by study region (the number of studies appears in parentheses)

In addition to VO_2max_, the following indicators of CRF were included in the selected studies: maximal physical working capacity (PWC) in Watts, PWC in Watts at variable and fixed HR thresholds (e.g., PWC at 75% of the predicted maximal heart rate or PWC at a heart rate of 170 beats per minute [PWC170]), time in seconds to HR threshold (e.g., exercise test duration to reach an HR of 130 beats per minute), energy expenditure in metabolic equivalents, and total exercise duration (in minutes or seconds). Eleven studies reported results for multiple measures of CRF. Almost two-thirds (48) of the studies applied maximal exercise testing (symptom limited), and one-third (27) applied submaximal testing. The treadmill (44) and the cycle ergometer (34) were commonly used exercise test machines in the included studies. Whereas the majority of European studies utilized cycle ergometers (76%), almost all studies conducted in North America used treadmills for exercise testing (94%; see Fig. 5).

### Exposure

#### Sociodemographic Characteristics

Table 2 summarizes the adjusted associations between CRF and potential correlates or determinants. Unsurprisingly, the most studied individual correlate of CRF was age. More than 80% of the samples found a negative association with CRF, indicating that fitness declined with age. Three samples found no significant relation between age and CRF. A positive association was reported for two samples, but the age ranges were relatively narrow in these studies (21 to 43 years [12] and 18 to 30 years [12, 59]).

**Table 2:** Summary of individual correlates and determinants of cardiorespiratory fitness

	Related to CRF		Unrelated to CRF	Summary of associations				
	Reference numbers	Association (+/−)	Reference numbers	#	#+	#−	#0	Association (+/−)
Sociodemographic characteristics								
Age	23, (12m^36^, 59m^36^)	+	(12f^15^,59f^17^), *60f*, 68m	43	2	38	3	− −
	3m, 5m, 13f, (14, 67), 16f, 16m, *17m*, 18, *22*, 24, 27f, 27m, 28f, 29f, 29m, 30m, 32m, 33, 35m, *37f*, *37m*, 39f, 40, 45m, 47m, *53f*, 54f, 54m, 56m^16^, *60m*^18^, 61m, 65f, 65m, 68f, 69f, 69m, 72f, 72m	−						
Sex (women vs. men)	(14, 67), *22*, 24, 33, 40, 18	−		6		6		−
Ethnicity	19f, 12f, 12m^37^, 14f		14m	5				n/a
Marital status (married vs. single)	45m	+		1	1			n/a
Socioeconomic status								
Education	*6f*, (12m^5^, 59m^7^), (12f^5^, 59f), 23m, 45m, 58^6^	+	*7f*, *7m*, (14m, 67), (14f, 67)	10	6		4	+
Parental education	*15* ^*38*^	−		1		1		n/a
Maternal education	*15* ^*39*^	+		1	1			n/a
Paternal education	*15* ^*40*^	−		1		1		n/a
Composite SES index	20f, 58	+	20m	3	2		1	+
Occupation	58, 45m^14^	+		2	2			n/a
Employment	45m	+		1				n/a
Working mother			6m	1			1	n/a
Income	45m	+		1	1			n/a
Financial strain	58^13^	+		1	1			n/a
Anthropometric measures								
Body mass index	10m, 13f, 16f, 18, 23m, *37m*, *37f*, 59m, 59f, *64m*^1^, 65m, 65f, 66m, 66f, 77m, 77f, 78m, 78f	−	*7f*, *7m*, 16m	24	3	18	3	−
	32m, 40, 56m	+						
Overweight	*63f*	−	67	2		1	1	n/a
Obese	*63f*, 67	−		2		2		n/a
Relative weight	11m, 11f	−		2		2		n/a
Body shape			3m	1			1	n/a
Waist circumference	13f, (14m, 77m), 16m, 16f, 23m, 30m, 30f, 78m, 78f	−	(77f, 14f)^8^	10		9	1	− −
Waist-to-hip circumference ratio	47m	−		1		1		n/a
Weight	6m	−		4	3	1		+
	24, 35m, 47m	+						
Body fat (%)	10m, 33m, 33f, 39f, 61m, 68m, 68f^2^	−		7		7		+
Total fat mass	72f, 72m	−		2		2		n/a
Lean mass (%)	9, *22*	+		2	2			n/a
Total lean mass	72m, 72f	+	9	3	2		1	+
Skeletal muscle mass	54m, 54f	+		2	2			n/a
Sum subscapular	12m, 12f	−		2		2		n/a
Appendicular lean mass/height squared			9	1			1	n/a
Appendicular lean mass (%)	9	+		1	1			n/a
Height	35m	+	10m	2	1		1	n/a
Birth weight	*64m*	+		1	1			n/a
Vital parameters								
Heart rate, resting	10m, 11m, 11f, 47m, 56m, 67	−		6		6		−
Heart rate, maximal exercise	47m	+		1	1			n/a
Heart rate, after CRF test	66m, 66f^4^	−		2		2		n/a
Mean blood pressure	56m, 68	−		2		2		n/a
Systolic blood pressure	12m, 12f, 77m	−	77f	4		3	1	−
Diastolic blood pressure	77m, 77f	−		2		2		n/a
Forced expiratory volume in 1 s	47m, 59m^3^, 59f^3^	+		3	3			+
Vital capacity	11m, 11f, 51	+		3	3			+
Aortic augmentation index	10m	−		1		1		n/a
Comorbidities and medications								
Coronary heart disease	47m	−		1		1		n/a
Asthma	47m	−		1		1		n/a
Hypertension			67	1			1	n/a
Diabetes			67	1			1	n/a
Beta-blocker use	30f, 30m	−		2		2		n/a
Shortness of breath upon exertion			32m	1			1	n/a
Biomarkers								
Bicarbonate	1	+		1	1			n/a
Anion gap	1	−		1		1		n/a
Vitamin D	4	+		1	1			n/a
(High-sensitivity) C-reactive protein	2, 41, 44f, 44m	−	67	5		4	1	−
Thyroid-stimulating hormone	36^9^			1				n/a
Hemoglobin	47m	+		1	1			n/a
Fasting serum insulin	47m	−	24	2		1	1	n/a
2-h glucose tolerance test			24	1			1	n/a
Glucose, mg/dL			77m, 77f	2			2	n/a
HbA1c, %			77m, 77f	2			2	n/a
Ferritin	50m^10^	−		1		1		n/a
High-density lipoprotein (HDL) cholesterol	56m^11^, 77m, 77f	+		3	3			+
Non-HDL cholesterol	56m^12^	+		1	1			n/a
Cholesterol	77f	−	(67, 77m)	2		1	1	n/a
Triglycerides	77m^42^		77f	2			1	n/a
Creatinine excretion	21f, 21m	+		2	2			n/a
White blood cell count			67	1			1	n/a
Homocysteine	43f	−	43m	2		1	1	n/a
Insulin-like growth factor (IGF) I			25f, 25m	2			2	n/a
IGF-binding protein 3 (IGFBP-3)			25f, 25m	2			2	n/a
IGF-I and IGF-I/IGFBP-3 ratio			25f, 25m	2			2	n/a
Nonesterified fatty acid			24	1			1	n/a
Red cell distribution width	9	−		1		1		n/a
Flow-mediated dilation			26	1			1	n/a
Nitroglycerin-mediated dilation	26^33^	+		1	1			n/a
Activity parameters								
Subjective measurements								
Overall PA								
PA index, various	11f, 11m, (12f, 59f), (12m, 59m), 33f, 33m, *37f*, *37m*, *38*, 48m, 49m, 56m, *60f*^29^, *60m*^29^	+	10m, 49f, 65f, 65m, *76m*	19	14		5	+
PA level, high vs. low	*73f* ^32^	+	49f, 49m	3	1		2	
Failure to meet PA recommendation			*63f*	1			1	n/a
PA times/week	23m, 47m, 66f, 66m	+		4	4			+
PA duration (hours/week)	45m	+		1	1			n/a
PA (in METS or MET minutes/week)	(14f, 67^30)^, (14m, 67^30)^, *38*, (45m, 47m), 68m^31^, *70*	+	68f	7	6		1	+
Energy expenditure (kcal/week)	45m	+		1	1			n/a
Moderate-to-vigorous PA	30f, 30m	+		2	2			n/a
Moderate PA	39f	+	75	2	1		1	n/a
Vigorous PA	75	+		1	1			n/a
Proportion of vigorous PA/all PA	14f, 14m	+		2	2			n/a
LTPA								
Regular exercise			32m	1			1	n/a
Activity > 2 h/week	35m	+		1	1			n/a
Training time (hours/week)	16f, 16m	−		2		2		n/a
Leisure sports activities (yes vs. no)	7f, 74m	+		2	2			n/a
LTPA, quartiles	22	+		1	1			n/a
Intensity of LTPA (in METS)	61m	+		1	1			n/a
LTPA, duration/day			61m	1			1	n/a
Energy expenditure during active leisure time	48	+		1	1			n/a
Caloric expenditure in sports activity	16f, 16m	+		2	2			n/a
Membership in a sports club			*7m*	1			1	n/a
Past participation (years of vigorous or moderate sporting activities)			49f, 49m	2			2	n/a
Occupational PA								
Occupational PA	74m	+	45m	2	1		1	n/a
Sedentary PA								
Sedentary PA	39f	−		1		1		n/a
Other PA measures								
Satisfied with sports performance (yes)			*7f*, *7m*	2			2	n/a
Positive attitude toward swimming			*6m*	1			1	n/a
Positive attitude toward soccer and handball			*7f*, *7m*	2			2	n/a
Positive attitude toward aerobic fitness			*7f*, *7m*	2			2	n/a
Objective measurements								
Accelerometer								
PA volume			52f	1			1	n/a
PA intensity	52f	+		1	1			n/a
Step count	13f	+		1	1			n/a
Moderate-to-vigorous PA (continuous)	13f, 18, 42f, 42m	+		4	4			+
Vigorous PA (continuous)	13f	+		1	1			n/a
Vigorous PA (none vs. any)	18			1	1			n/a
Sedentary PA	42f, 42m	−		2		2		n/a
Physical fitness								
Knee extension torque			9	1			1	n/a
Handgrip strength			9	1			1	n/a
Bench press	*7m*	+	*7f*	2	1		1	n/a
Sargent jump	*6f*	+		1	1			n/a
Other								
Physical activity energy expenditure	24	+		1				n/a
Other health behaviors								
Smoking vs. non-smoking	*8f*, *8m*, 11f, 11m, *22*, 23m, 30m, 32m, (*37f*, *63f*), *37m*, *55m*, *60m*^22^, *60f*^22^	−	14m, 16f, 16m, 24, 30f, 35m, (12f^19^, 59f^21^), ( 12m^19^, 59m^21^)	22	1	13	8	−
	(14f, 67)^41^	+						
Number of cigarettes	56m^20^, 68m^23^	−	68f	3		2	1	−
Alcohol	12f , 57^24^	+	12m^27^	3	2		1	+
Carbohydrates (g/day)	47m	+		1	1			n/a
Diet quality score	57f, 57m^35^	+		2	2			n/a
Meat dietary pattern	57f^26^, 57m^26^	−		2		2		n/a
Fruit–vegetable dietary pattern	57m^28^	+	57f	2	1		1	n/a
Childhood television viewing	(*31*, *46*)	−		1		1		n/a
Adult television viewing	(*31*, *46*)	−		1		1		n/a
Sleep problems	62^25^	−		1		1		n/a
Other								
Commuting distance	34^34^	−		1		1		n/a
Gestational age (mother)	*64m*	+		1	1			n/a
Attachment loss (dental)			67	1			1	n/a
Probing depth (dental)			67	1			1	n/a
Preterm birth			*71*	1	1			n/a

+: positive association; −: negative association; 0: null association; n/a: summary measure not applicable because the number of independent samples investigating the relationship is less than three. The numbers in the summary table refer to the reference number for each study. f: women only; m: men only. Samples from studies with longitudinal designs are marked in italic. Separate studies drawing on the same study population and reporting redundant exposures are presented in parentheses and were counted as one unit of analysis. *CRF* cardiorespiratory fitness, *MET* metabolic equivalent, *LTPA* leisure-time physical activity, *PA* physical activity, *PWC* physical working capacity

^1^Inverse U-shaped association

^2^Negatively associated with annual change in VO_2max_

^3^Significant association with test duration but not with work load 130

^4^Significant association with time to heart rate 130 but not with test duration

^5^Significant association with test duration but not with time to HR130

^6^Inverse U-shaped association (medium > high > low)

^7^Significant association with test duration but not with WL130

^8^Women with low CRF showed significantly higher waist circumference, compared with women with high CRF, in [77] (adjusted for “race” and age). Using the same data, in [14], women with high waist circumference did not show significantly different levels of CRF, compared with women with normal WC, after adjustment for multiple variables

^9^Lower VO_2peak_ in the second quintile than in the third quintile of thyroid-stimulating hormone

^10^Lower odds of high fitness (VO_2max_) with elevated serum ferritin (> 300 ng/ml) vs. non-elevated serum ferritin (< 300 ng/ml)

^11^Significant association with PWC150/kg but not with PWC150, workload/heart rate, test duration, or workload

^12^Significant association with workload and workload/heart rate but not with PWC150, PWC150/kg, or test duration

^13^Higher METS with medium vs. low financial strain

^14^Higher mean VO_2max_ among white-collar workers than among blue-collar workers and farmers

^15^Significant positive association with time to heart rate 130; significant negative association with test duration

^16^Significant association with workload, workload/heart rate, PWC150, and test duration but not with PWC150/kg

^17^Significant negative association with test duration only for black women; significant positive association with WL130 only for white women

^18^Significant association with exercise test duration only for white men (not for black men)

^19^Significant negative association with test duration; significant positive association with time to heart rate 130

^20^Significant negative association with workload, test duration, and workload/heart rate; no significant association with PWC150; significant positive association with PWC150/kg

^21^Significant negative association with test duration; significant positive association with PWC130

^22^Significant negative association with test duration for black men and black women; significant negative association with WL130 for white men and black women

^23^Significant negative association with annual change in VO_2max_

^24^Significant positive association with test duration for beer and wine but not for liquor

^25^Significant positive association of VO_2max_ with repeated awakenings and daytime sleepiness but not with sleep initiation problems or early awakening

^26^Significant negative association of test duration with meat dietary pattern for white men and women

^27^Significant negative association with test duration; significant positive association with time to heart rate 130

^28^Significant positive association of test duration with fruit–vegetable dietary pattern for white men

^29^Significant positive association with test duration for all subgroups; significant positive association with PWC130 only for white men and white women

^30^Significant positive association for high vs. none activity in METmin/week

^31^Significant positive association with VO_2max_ at first checkup

^32^Persistently active vs. persistently inactive; no significant association in other categories vs. persistently inactive

^33^Significant positive association only among non- or ex-smokers (not in current smokers)

^34^Significant negative association only for continuous measure of commuting distance and for 11–15 miles vs. 0–5 miles

^35^Significant positive association of test duration with a priori diet quality score for white men

^36^Significant positive association with time to heart rate 130; no significant association with test duration

^37^Significant positive association with test duration; no significant association with time to heart rate 130

^38^Significant higher risk of fitness decrease and lower risk of fitness persistence for medium vs. low parental education

^39^Significant higher risk of fitness persisting for high vs. low maternal education

^40^Significant lower risk of fitness persisting for medium vs. low parental education

^41^CRF was positively associated with smoking for both sexes in [67]. In [14], there was a positive association with smoking for women but not for men

^42^Significantly higher values for medium vs. high fitness but not for low vs. high fitness

Sex differences were investigated in six studies, all of which reported significantly higher CRF among men than among women.

The association between CRF and ethnicity was investigated in five samples, all in the USA. In four samples, higher CRF was observed among “whites” than among “blacks,” whereas one sample of men observed no association between ethnicity and CRF.

We identified 19 samples investigating the associations between CRF measures and socioeconomic status (SES) variables. The most common SES measure was education (ten samples), and a positive association between education and CRF was reported for a majority of the samples, although in some samples no significant association was observed. Composite SES indexes were investigated in three samples, with summary measures also showing a positive association with CRF.

#### Anthropometric Measures

Anthropometric measures were frequently investigated in the included studies. BMI, the most frequently analyzed anthropometric factor (24 samples), showed an inverse association with CRF, as did WC and body fat. Weight and total lean mass were positively associated with CRF in at least 60% of the samples.

#### Vital Parameters

Among studies investigating the relationship between vital parameters and CRF, resting HR, systolic BP, forced expiratory volume (FEV) in 1 s, and vital capacity were each studied in three or more samples. Resting HR and systolic BP showed negative associations with CRF, whereas vital capacity and FEV in 1 s were positively associated with CRF.

#### Biomarkers

A variety of biomarkers were investigated in a total of 22 samples. Most clearly, CRP showed a negative association with CRF. High-density lipoprotein (HDL) cholesterol was found to be positively associated with CRF in three samples. Other biomarkers were studied in fewer than three samples.

#### Activity Parameters

About half of the studies (39) reported at least one measure of PA. A wide range of PA measures were used, but only five of these measures were reported in at least three independent samples, allowing the calculation of a summary measure. Most studies investigating activity parameters assessed subjective PA (e.g., via the International Physical Activity Questionnaire; 34), but objective activity parameters (using an accelerometer or objective physical fitness measures) were collected in four studies. Most studies assessing subjective PA used measures of overall PA, namely customized PA indexes, dichotomous PA level (high vs. low), PA times per week, or PA in metabolic equivalents/metabolic equivalent minutes. Forms of LTPA were also often investigated, but these were too diverse for the calculation of summary measures. Other specific domains of PA (transport PA, occupational PA, or household PA) and sedentary behavior were rarely or never examined. Moderate-to-vigorous PA was the only objective measure of PA (measured using an accelerometer) reported in at least three independent samples. Almost all summarized PA exposures showed a positive association with CRF. Only dichotomous PA level (high vs. low) showed no association (based on three samples).

#### Other Behavioral Factors

Other than PA, smoking status was the most frequently investigated indicator of health behavior (22 samples). Overall, smoking was inversely associated with CRF. However, in a considerable number of samples, smoking status was found to be unrelated to CRF, and one study found a positive association between smoking and CRF in women. The number of cigarettes consumed, which was studied in three samples, was also inversely associated with CRF.

Alcohol consumption was examined in three samples. Whereas one mixed-sex sample showed a positive association between alcohol consumption and CRF, another study found a positive association among women but varying results depending on the CRF measure among men.

For all other investigated behavioral factors, the results could not be summarized because of a limited number of samples.

#### Sensitivity Analyses

No significant differences in the association with CRF were found for age, education, or WC when comparing different methods of CRF assessment (direct VO_2max_ vs. indirect VO_2max_ vs. other), different test machines used (bicycle ergometer vs. treadmill) or sex (men vs. women vs mixed samples; Additional file 2: Tables OR2.1 to OR2.19). For BMI, PA index, and smoking, we found differences in the results by the CRF measure used (*p* < 0.05, Fisher’s exact test) but not by sex and test device. The association between CRF and BMI did not differ between samples where adjustments for body weight measures were performed, compared with samples where this adjustment was not performed.

#### Update Literature Search

The limited update literature search in PubMed yielded 383 records. After de-duplicating for records found in the initial search and title and abstract screening, 55 records remained for full-text screening. A total of seven records did fulfil the eligibility criteria [158–164]. Study characteristics and results of these studies can be found in Additional file 3: Table OR3.1. Overall, results from these studies are in line with the reported results from the main search and only few new potential correlates or determinants were investigated: one study analyzing caffeine consumption found a positive association with CRF among women, but not among men [162]. Another study reported a negative association between various plasma fatty acids and CRF among men, but only between arachidonic acid and CRF among women [163]. Moreover, in a further study, a negative association between anemia and CRF among women and a negative association between estimated glomerular filtration rate and CRF among men was reported [161]. As these factors were reported in two or less samples, calculation of a summary measure was not possible.

## Discussion

### Summary of Evidence

This systematic review aimed to give a detailed overview of the potential individual factors influencing CRF and to analyze the consistency of the results of existing research on this topic. Overall, 3016 records were identified, and 78 articles were ultimately included. We found evidence that CRF decreases with age, is lower among women than among men, and is associated with ethnicity. CRF was positively associated with SES, FEV, and vital capacity, and negatively associated with BMI, weight, WC, body fat, resting HR, systolic BP, and CRP. As expected, CRF was associated with several measures of PA. Furthermore, CRF showed a negative association with smoking and a positive association with alcohol consumption in the majority of the included studies. Age, BMI, WC, PA index, smoking, and education were the most investigated factors (≥ 10 samples). For these factors, results showed no significant sex differences. To our knowledge, this is the first comprehensive review investigating the individual determinants and correlates of CRF. A review conducted by Ortega et al. [98] summarized the relationship between fitness and CVD risk factors among children and adolescents. Another systematic review focused on studies investigating the association between genetic variants and CRF trainability [113]; however, this was beyond the scope of the present review.

### Comparison with Other Studies and Interpretation of Results

#### Sociodemographic Characteristics

It is well established that CRF declines with age. This may be because of physiological adjustments, such as atrophy of muscle mass with biological aging, changes in lifestyle, or increasing disease burden and medication [118]. Although the decline in CRF with age is widely accepted, the causes behind this relation are not yet clearly understood [119].

The contradictory results for age found in two included samples can partially be explained by the limited age ranges of these study samples: The studies focused on young or middle-aged individuals. Because maximum CRF is usually attained between 20 and 30 years of age [37, 86, 120], linear age trends may be positive in young study populations. When the study population has an older age range, a decline in CRF with age becomes apparent—a trend that seems to be more pronounced among men than among women [22, 37, 120, 121]. However, these differences largely disappear when the decline in CRF is expressed as a percentage change rather than as an absolute value [37, 121]. Two studies used meta-analytical approaches to combine data and generate general age-specific reference values and predictive equations [99, 122]. However, because of a high level of heterogeneity in the assessment of CRF, as well as differences in methods and study population characteristics, these results should be interpreted with caution [123]. Furthermore, the effect of PA on the decline in CRF over the life course is unclear [120, 121]. Although meta-analyses of cross-sectional data have not reported evidence that increased PA levels mitigate the decline [124, 125], longitudinal studies have found that individuals with enhanced PA have less decline in CRF per decade than do sedentary individuals [120]. Independent of age, a longitudinal study among men found a greater decline in CRF over time associated with a greater risk of total mortality [157].

There is clear evidence that, overall, compared with men, women have lower CRF levels. The difference between men and women is often estimated at around 20% [20, 126–128]. Common physiological explanations for this difference are women’s smaller body and organ size and higher body fat percentage, compared with men [100]. Correspondingly, Fleg et al. [22] pointed out that, when VO_2max_ is expressed relative to muscle mass (e.g., ml/kg muscle mass/min), the difference between the sexes is often eliminated. In addition to physiological explanations, there are differences in behavioral and social factors [100] (e.g., differences in PA behavior) that could partly explain the CRF differences between men and women.

Higher SES is predominantly associated with higher CRF. Although our summary measures could only be calculated for education and composite SES indicators, no inverse association was reported for the other SES indicators, which were investigated in fewer than three samples. A positive association between education and CRF was previously confirmed in a meta-analysis that included 9435 adults from four population-based studies [102]. These findings are also in line with systematic reviews investigating the association between LTPA and CRF [129]. Because aerobic LTPA is strongly associated with CRF, lower LTPA levels among lower-SES population groups may contribute to lower CRF levels in these groups. Lower SES is not only associated with lower aerobic LTPA but also with higher fat and sugar consumption, lower consumption of fruits and vegetables [130], and higher smoking prevalence [131]. In addition to unfavorable health behaviors, major NCD risk factors like obesity [132] and chronic diseases such as diabetes and CVD are more prevalent among population groups with lower SES. Thus, unfavorable health behaviors and risk factors that may be associated with CRF potentially reinforce the effect of SES on CRF. However, these associations have been confirmed mainly in high- and upper-middle-income countries, and the situation in low- or middle-income countries might be different because these countries are in earlier stages of the epidemiological transition [133]. High-SES population groups in these countries may move to the next stage before low-SES groups and therefore also adopt unfavorable health behaviors earlier. Consider, for example, the case of obesity: In low-income countries, the more affluent are more likely to be obese [134].

#### Anthropometric Measures and Vital Parameters

Anthropometric measures were often investigated in the included studies, showing a clear inverse association between BMI and CRF. Most samples with an inverse association between CRF and BMI used a body weight-adjusted measure of CRF (such as relative VO_2max_ in ml/kg/min) that took into account the differences in CRF because of BMI. We performed sensitivity analyses to check whether the association between CRF and BMI differed depending on the adjustment of measures for relative body weight and found no significant differences. Although CRF and BMI are related, a high BMI does not necessarily mean a low fitness level. High muscle mass can also lead to having a high BMI caused by the dense structure of muscle mass. Research suggests that, compared with the health risks of having a high BMI, having low CRF is a more important risk factor: a systematic review found that the risk for cardiovascular mortality was lower among individuals with high BMIs and high levels of CRF, compared with those with normal BMIs and low levels of CRF [135]. Furthermore, studies suggest that individuals with low CRF have higher levels of general and abdominal adiposity, as measured by WC, for example, than do individuals with moderate/high CRF, independent of BMI [136]. Thus, it is not surprising that WC was negatively associated with CRF in almost all of the included studies. Likewise, percentage of body fat was found to be negatively associated with CRF in all samples, and lean body mass (or skeletal muscle mass, which accounts for most of the lean mass) was positively associated with CRF. Again, it should be noted that the effects of these factors are strongly depending on the application and procedures (e.g., relative to body weight, relative to fat free mass, or relative to predicted weights) of data normalization for the CRF measure [165].

All of the reviewed studies demonstrated the expected associations between CRF and vital capacity parameters, most of which have been extensively studied in endurance training intervention studies and well documented in the exercise physiology literature [137]. VO_2max_ is one of many physiological parameters that improve as a result of endurance training. Increased cardiorespiratory capacities such as stroke volume and vital capacity, as well as improved muscle and blood composition, contribute to a higher maximal oxygen uptake. These adaptations lead to a more efficient supply of energy and oxygen for bodily functions and to a reduction in resting HR and BP.

#### Behavioral Factors

The majority of the included studies found a negative association between CRF and smoking. These findings may be explained by multiple pathways. First, tobacco smoking may trigger a cascade of modifications in the respiratory organs, which can lead to reduced pulmonary function. Limited pulmonary function, in turn, may be negatively associated with CRF [138]. Second, because CRF and PA are closely related and PA is also inversely associated with smoking, negative health behaviors such as inactivity, smoking, and alcohol consumption may often cluster together [139], and (in)activity acts as a confounder in the relation between smoking and CRF. Third, the relation between CRF and smoking also may be explained by SES because both smoking [131] and low fitness [102] seem to be associated with low SES.

Because the observed association of CRF with alcohol is based on only three samples, these findings should be interpreted with caution. One study found a direct association with some types of alcohol (e.g., wine) but not others (e.g., liquor) [57]. A recent investigation found better fitness among moderate drinkers, compared with heavy drinkers and abstainers, using data from five population-based studies [101]. Although there are potential explanations for the U-shaped relation between alcohol intake and CRF [101], it is also possible that this is a result of confounding [140].

Dietary risk factors, such as a diet high in sugar-sweetened beverages, are known to be related to cardiometabolic risk factors [141], but relatively little is known regarding the relationship between these dietary factors and CRF [57, 70]. Recent research suggests that CRF may act as a mediator of the relation between macronutrient intake relative to weight and fat mass in adolescents: individuals with a higher macronutrient intake, which may be related to engaging in more PA, may suffer from obesity less often, especially when they have high CRF levels [142].

The relation between PA and CRF has been widely discussed in the literature, and the positive impacts of PA and CRF on health are well documented [143]. CRF is influenced by several physiological traits, especially the performance of the cardiovascular system. Thus, in the relationship between PA and CRF, PA is the modifiable variable: performing PA allows individuals to reach their highest possible level of CRF, which is also determined by heredity and other factors [84, 144]. Although the nature of the association between PA and CRF is widely accepted, it cannot be ruled out that low CRF levels caused by genetic pre-conditions can lead to lower PA levels [84].

The results of this systematic review reflect the well-established relation between CRF and PA. A wide variety of objective and subjective PA measures were used in the included studies, and a majority of these studies found a significant positive association between PA and CRF. Whereas overall PA and LTPA were commonly investigated, only a few studies investigated other domains such as occupational PA. Other domain-specific types of PA may have different effects on health. For example, studies have shown that occupational PA can either have no association or be negatively associated with health outcomes, whereas LTPA affects health in a strictly beneficial way [145, 146]. While conducting this review, we found only two studies that investigated the relation between PA at work and CRF, and these studies had varying results. One study found no association between CRF and occupational PA but a significant positive association between CRF and hours of PA per week [45]. The other study found a positive association for both LTPA and occupational PA [74]. Considering their potentially varying effects on health, the associations between domain-specific types of PA and CRF should be further examined.

Overall, health habits such as dietary, smoking, and PA behaviors are usually measured with considerable inaccuracy. This could mean that there is imprecision in the measurement of health behaviors in studies investigating these factors as correlates and determinants of CRF [47].

### Strengths and Limitations

To our knowledge, this is the first systematic review investigating the individual correlates and determinants of CRF. The semi-quantitative approach used in this review made it possible to present a concise summary of the results from 78 studies and to provide a comprehensive overview of the current state of research and potential research gaps.

We acknowledge that such a comprehensive review also has limitations. The majority of the studies included in this review were cross-sectional; therefore, statements about causal relationships between exposures and outcomes are limited. Furthermore, not all studies used VO_2max_ (the “gold standard”) as an outcome, diminishing the comparability of results across the studies. Although other methods for measuring CRF (e.g., exercise test duration) have been shown to be correlated with VO_2max_ [147, 148], differences in methods could lead to deviating results. It is interesting to note that the included studies that reported the associations between an exposure and various measures of CRF often found similar associations, independent of the measurement method used. Several studies also obtained CRF results using submaximal exercise-based predictive equations. Although it has been found that the results of assessed and predicted CRF can vary [111], a systematic review has demonstrated that these predictive equations are quite accurate [149]. In addition to differences in the method of measurement (indirect vs. direct), different indicators of CRF (e.g., VO_2max_ vs. others) were not taken into account when discussing the results. This could be considered a potential bias because sensitivity analyses showed significant differences in the association between directly assessed VO_2max_, indirectly assessed VO_2max_, and other CRF measures for some of the exposures. Variations in the methods used in the statistical analyses may also have affected the results. In this systematic review, we only analyzed the adjusted results from multivariable analyses. Because fewer variables usually reach the defined level of significance in such analyses, compared with univariate analyses [115], a bias toward null findings is possible [150]. Such a bias may be exacerbated by the use of the fully adjusted models.

Because of the wide range of correlates and determinants analyzed, as well as the significant heterogeneity in analytical methods and study samples, it was not feasible to conduct a meta-analysis. Correspondingly, we were unable to show the strength of the associations (effect measures) or to adjust the results for sample size. In addition, variation in adjustment for confounding among the studies was not considered in the interpretation of the results for each outcome, although this difference may have led to divergent results across the studies. Due to restrictions in the search strategy and the intra-study requirements to participate in CRF testing, the study populations usually consist of a relatively healthy population. However, it cannot be ruled out that some of the reported associations are influenced by underlying chronic diseases. Furthermore, the universal perspective of this review did not allow determinants, correlates, mediators, moderators, and confounders to be differentiated [151]. However, the focus of this review was to show the consistency of reported associations. Certain indicators may be underrepresented for specific reasons (e.g., underreporting or limitations of the search strategy). Existing studies have shown that correlates and determinants may differ across the life course [37, 98]. Future studies should investigate whether the results of this systematic review can be confirmed for young people and older adults. Furthermore, although this was beyond the scope of the present review, environmental and interpersonal determinants probably also play an important role in determining or moderating CRF and should be further investigated [103].

## Conclusions

Our comprehensive systematic review showed that there is a broad range of individual factors associated with CRF. Whereas factors such as age, BMI, and WC showed consistent evidence of an association with CRF, other factors showed conflicting or insufficient evidence of an association. For example, few studies have investigated the relationship between CRF and behavioral factors other than PA and smoking, or the association between CRF and psychosocial factors.

Several implications for health promotion practice and for future research can be drawn from this review. First, sociodemographic factors shown to be associated with CRF in this review can be used to help identify subgroups of relatively unfit individuals (e.g., people with low education) who should be targeted for interventions. Second, the strong association between aerobic LTPA and CRF was confirmed in this review, but less is known about the relation between CRF and domain-specific PA. Thus, future studies should compare the impact of domain-specific PA on CRF. The comparability of the results was hampered by differences in the assessment of PA; therefore, the use of standardized domain-specific measures, such as the Global Physical Activity Questionnaire [152] or the European Health Interview Survey–Physical Activity Questionnaire [153], is recommended. In addition, sedentary behavior plays an important role in the global strategy to tackle NCDs, but little is known about the association of this factor with CRF in adults [154]. Third, there is some evidence that health behaviors other than PA, such as smoking, alcohol consumption, and nutrition, are also associated with CRF. Thus, multi-behavioral interventions might be an appropriate approach when implementing preventive measures to enhance health status in the general adult population [155], although the effects of these approaches have been found to be limited [156].

Future reviews could build on the results of the present systematic review by consolidating the evidence regarding the correlates and determinants of CRF in younger and older population groups, which were not included in this review. Moreover, evidence from low- and middle-income countries is needed to improve the generalizability of the results because most of the studies included in this review were conducted in high-income country settings. Notwithstanding the close association between CRF and PA and the fact that CRF is partly genetically determined, relatively little is known about the complex interplay among the potential determinants of CRF. This review can be a first step toward the development of a comprehensive model of (cardiorespiratory) fitness that integrates not only physiological aspects but also a broad set of socio-ecological factors.

## Additional Files

Additional file 1: Quality Assessment. (PDF 490 kb)

Additional file 2: Sensitivity Analyses. (PDF 253 kb)

Additional file 3: Limited Search Update. (PDF 210 kb)

Additional file 4: Data extraction table. (XLSX 215 kb)

## Data Availability

All data generated or analyzed during this study are included in this published article (and its supplementary information files).

## Abbreviations

BMI: Body mass index
BP: Blood pressure
CRF: Cardiorespiratory fitness
CRP: C-reactive protein
CVD: Cardiovascular disease
HDL: High-density lipoprotein
HR: Heart rate
LTPA: Leisure-time physical activity
NCD: Non-communicable disease
PA: Physical activity
PRISMA: Preferred Reporting Items for Systematic Reviews and Meta-Analyses
PROSPERO: International Prospective Register of Systematic Reviews
SES: Socioeconomic status
VO_2max_: Maximal oxygen consumption
VO_2peak_: Peak oxygen consumption
WC: Waist circumference
