# Supplementary material for: Correlates and Determinants of Cardiorespiratory Fitness in Adults: a Systematic Review
Source: Sports Med Open. 2019 Sep 3;5:39. doi: 10.1186/s40798-019-0211-2 (PMC6722171; doi:10.1186/s40798-019-0211-2)
Supplement: Supplementary file 1 — Quality Assessment. (PDF 490 kb) [file 40798_2019_211_MOESM1_ESM.pdf]

Online Resource 1: Quality Assessment

Correlates and determinants of cardiorespiratory fitness in adults: a systematic review

Johannes Zeiher*, Katherine J. Ombrellaro, Nita Perumal, Thomas Keil, Gert B. M. Mensink, Jonas D. Finger

*** Corresponding author:**
Johannes Zeiher
Unit of Health Behaviour
Department of Epidemiology and Health Monitoring
Robert Koch Institute
General-Pape-Straße 62-66
12101 Berlin, Germany
Email: zeiherj@rki.de

- Each category was scored ‘yes’=1, ‘no’=0, ‘not reported (NR)’=0 or ‘not applicable’=not taken into account for calculation
- Underlined categories were multiplied by a factor of 1,5
- Final assessment was calculated by sum of scores divided by maximal score.
- Risk of bias was categorized as high when < 49% requirements met, moderate when 50–75% requirements met, and low when >75% requirements met

Categories:

1. Was the research question clearly stated?
2. Were the correlates of cardiorespiratory fitness being investigated clearly stated?
3. Was the outcome of cardiorespiratory fitness clearly stated?
4. Was the study population clearly defined?
5. Were the participants sampled using a probability-based sampling strategy?
6. Was the sampling frame at a national level?
7. If participants were selected from clusters, were the number of clusters ≥ 50?
8. Were the inclusion and exclusion criteria for being in the study pre-specified?
9. Was the response rate of eligible participants >50%?
10. Was loss to follow-up in cohort studies after baseline 20% or less?
11. Where applicable, were the correlate(s) of cardiorespiratory fitness and potential confounders objectively measured through validated instruments?
12. Was the outcome variable clearly defined, valid, reliable, and reliable?
13. Was cardiorespiratory fitness measured using an objective, reliable, and validated methodology?
14. Was cardiorespiratory fitness measured consistently for all participants?
15. Were key potential confounding variables measured and adjusted for statistically to account for their impact on the relationship between the exposure and outcome variables (Age, sex, PA, measure of body weight)? *[factor 1,5]*
16. Did the study investigate interaction between exposure variables?
17. Was any sensitivity analysis conducted?
18. Did authors report their funding source and/or make a statement regarding conflicts of interest?

Table OR1.1 Customized risk of bias assessment

	Ref.[1]	1	2	3	4	5	6	7	8	9	10	11	12	13	14	15	16	17	18	Final Assessment	Risk Evaluation
**Abramowitz**	[1]	1	1	1	1	1	1	1	1	1	NA	1	1	1	1	1,5	1	1	1	100,0%	Low
**Agarwal**	[2]	1	1	1	1	1	1	1	1	1	NA	1	1	1	1	0	0	1	1	85,7%	Low
**Allen**	[3]	1	0	1	1	0	0	0	0	NR	NA	1	1	1	1	0	0	0	0	40,0%	High
**Ardestani**	[4]	1	1	1	1	NR	NR	NR	1	NR	NA	1	1	1	1	1,5	1	1	1	77,1%	Low
**Babcock**	[5]	1	1	1	1	0	0	0	1	NA	NA	1	1	1	1	0	0	1	1	62,9%	Moderate
**Barnekow-Bergkvist 1998**	[6]	1	1	1	1	0	1	NA	1	NR	0	1	1	1	0	0	0	0	1	54,1%	Moderate
**Barnekow-Bergkvist 2001**	[7]	1	1	1	1	0	1	NA	1	NR	0	1	1	1	0	0	0	0	1	54,1%	Moderate
**Bernaards**	[8]	1	1	1	1	0	0	0	1	NR	NR	1	1	1	1	1,5	1	1	1	73,0%	Moderate
**Bijlsma**	[9]	1	1	1	1	0	0	0	1	NR	NA	1	1	1	1	0	0	1	1	62,9%	Moderate
**Binder**	[10]	1	1	1	1	0	0	0	1	NR	NA	1	1	1	1	0	0	1	1	62,9%	Moderate
**Blair**	[11]	1	1	1	1	0	0	0	1	NR	NA	1	1	1	1	0	1	1	0	62,9%	Moderate
**Braun**	[12]	1	1	1	1	1	1	1	1	1	NA	1	1	1	1	0	1	1	0	85,7%	Low
**Cao**	[13]	1	1	1	1	0	0	0	1	NR	NA	1	1	1	1	0	0	1	1	62,9%	Moderate
**Ceaser**	[14]	1	1	1	1	1	1	1	1	1	NA	1	1	1	1	1,5	0	1	1	94,3%	Low
**Cleland**	[15]	1	1	1	1	1	1	1	1	0	0	1	1	1	1	0	0	1	1	75,7%	Low
**Dagan**	[16]	1	1	1	1	0	0	0	1	NA	NA	1	1	1	1	1,5	1	1	1	77,1%	Low
**Dehn**	[17]	1	1	1	1	0	0	0	0	NR	NA	1	1	1	1	0	0	1	1	57,1%	Moderate
**Dyrstad**	[18]	1	1	1	1	0	1	0	1	NR	NA	1	1	1	1	1,5	0	1	1	77,1%	Low
**Farrell**	[19]	1	1	1	1	0	0	0	1	NR	NA	1	1	1	1	0	0	0	0	51,4%	High
**Finger**	[20]	1	1	1	1	1	1	1	1	1	NA	1	1	1	1	1,5	1	0	1	94,3%	Low
**Fleg 1988**	[21]	1	1	1	1	0	0	0	1	NA	NA	1	1	1	1	0	0	1	NR	57,1%	Moderate
**Fleg 2005**	[22]	1	1	1	1	0	0	0	1	NA	NA	1	1	1	0	0	1	1	NR	57,1%	Moderate
**Fogelholm**	[23]	1	1	1	1	0	1	0	1	1	NA	1	1	1	1	0	1	1	1	80,0%	Low
**Franks**	[24]	1	1	1	1	1	1	NR	1	NR	NR	1	1	1	1	0	1	1	1	75,7%	Low
**Gläser 2010**	[25]	1	1	1	1	1	1	NR	1	1	NA	1	1	1	1	1,5	0	1	1	88,6%	Low
**Gläser 2011**	[26]	1	1	1	1	1	1	NR	1	1	NA	1	1	1	1	0	1	1	1	85,7%	Low
**Grigaliuniene**	[27]	1	0	1	1	0	0	0	1	NR	NA	1	1	1	1	0	0	0	0	45,7%	High
**Gulati**	[28]	1	1	1	1	0	1	0	1	NR	NA	1	1	1	1	0	0	0	1	62,9%	Moderate
**Habedank**	[29]	1	1	1	1	0	0	0	1	NR	NA	1	1	1	1	0	0	1	1	62,9%	Moderate
**Hakola**	[30]	1	0	1	1	1	1	NR	1	1	NA	1	1	1	1	0	0	1	1	74,3%	Moderate
**Hancox**	[31]	1	1	1	1	0	1	NR	1	1	1	1	1	1	1	1,5	0	1	1	83,8%	Low
**Hawari**	[32]	1	1	1	1	0	0	0	1	NR	NA	1	1	1	1	1,5	0	0	1	65,7%	Moderate

**Heil**	[33]	1	0	1	1	0	0	0	1	NR	NA	1	1	1	1	1,5	0	1	1	65,7%	Moderate
**Hoehner**	[34]	1	1	1	1	0	0	0	1	NA	NA	1	1	1	1	1,5	1	1	1	77,1%	Low
**Inbar**	[35]	1	0	1	1	0	0	0	1	NA	NA	1	1	1	1	1,5	0	1	0	60,0%	Moderate
**Ittermann**	[36]	1	1	1	1	1	1	0	1	1	NA	1	1	1	1	1,5	0	1	1	88,6%	Low
**Jackson**	[37]	1	1	1	1	0	0	0	1	NR	NR	1	1	1	1	1,5	1	0	1	67,6%	Moderate
**Kemper**	[38]	1	1	1	1	0	0	0	1	NR	0	1	1	1	1	1,5	1	1	1	73,0%	Moderate
**Kim 2011**	[39]	1	1	1	1	0	0	0	0	NR	NA	1	1	1	1	0	0	0	1	51,4%	Moderate
**Koch**	[40]	1	1	1	1	1	1	0	1	1	0	1	1	1	1	0	1	1	1	81,1%	Low
**Kondo**	[41]	1	1	0	1	0	0	0	1	NR	NA	1	0	1	1	0	0	0	0	40,0%	High
**Kulinski**	[42]	1	1	1	1	1	1	0	1	1	NA	1	1	1	1	1,5	0	1	1	88,6%	Low
**Kuo 2005**	[43]	1	1	1	1	1	1	0	1	1	NA	1	1	1	1	1,5	1	1	1	94,3%	Low
**Kuo 2007**	[44]	1	1	1	1	1	1	0	1	1	NA	1	1	1	1	1,5	0	1	0	82,9%	Low
**Lakka**	[45]	1	1	1	1	1	0	0	NR	NR	NA	1	1	1	1	0	0	1	1	62,9%	Moderate
**Landhuis**	[46]	1	1	1	1	NR	0	NR	1	1	1	1	1	1	1	0	0	1	1	70,3%	Moderate
**Laukkanen**	[47]	1	1	1	1	1	0	0	1	0	NA	1	1	1	1	0	0	0	1	62,9%	Moderate
**Lefevre 2002**	[48]	1	0	1	1	1	1	NR	0	NR	0	1	1	1	1	0	0	1	1	59,5%	Moderate
**MacAuley**	[49]	1	1	1	1	1	1	NR	1	1	NA	1	1	1	1	1,5	0	0	1	82,9%	Low
**Mainous**	[50]	1	1	1	1	1	1	1	1	NR	NA	1	1	1	1	1,5	0	1	1	88,6%	Low
**Makrides**	[51]	1	1	1	1	0	0	0	1	NA	NA	1	1	1	1	0	0	1	1	62,9%	Moderate
**Nokes**	[52]	1	1	1	1	0	0	0	0	NA	NA	1	1	1	1	0	0	0	0	45,7%	High
**Plowman**	[53]	1	0	1	0	0	0	0	0	0	NA	1	1	1	1	0	0	1	1	45,7%	High
**Sanada**	[54]	1	1	1	1	0	0	0	NR	NA	NA	1	1	1	1	0	0	0	0	45,7%	High
**Sandvik**	[55]	1	1	1	1	0	0	0	1	1	NA	1	1	1	1	0	0	0	0	57,1%	Moderate
**Seccareccia**	[56]	1	0	1	1	0	1	0	1	1	NA	1	1	1	1	1,5	0	0	1	71,4%	Moderate
**Shikany**	[57]	1	1	1	1	1	1	1	1	1	NA	1	1	1	1	1,5	1	1	1	100,0%	Low
**Shmueli**	[58]	1	1	1	1	0	0	0	1	NA	NA	1	1	1	1	1,5	1	1	0	71,4%	Moderate
**Sidney 1992**	[59]	1	1	1	1	1	1	1	1	1	NA	1	1	1	1	1,5	0	1	1	94,3%	Low
**Sidney 1998**	[60]	1	0	1	1	1	1	1	1	1	NA	1	1	1	1	1,5	0	1	1	88,6%	Low
**Singhal**	[61]	1	1	1	1	0	0	0	1	NA	NA	1	1	1	1	1,5	0	0	1	65,7%	Moderate
**Strand**	[62]	1	1	1	1	NR	0	0	1	1	NA	1	1	1	1	1,5	1	1	1	82,9%	Low
**Sui**	[63]	1	1	1	1	0	0	0	1	NR	NA	1	1	1	1	1,5	0	0	1	65,7%	Moderate
**Svedenkrans**	[64]	1	1	1	1	0	1	0	1	NA	NA	1	1	1	1	0	0	0	1	62,9%	Moderate
**Talbot**	[65]	1	1	1	1	0	0	0	1	NR	NA	1	1	1	1	1,5	0	0	1	65,7%	Moderate
**Tammelin**	[66]	1	1	1	1	0	1	0	1	1	NA	1	1	1	1	0	0	1	1	74,3%	Moderate
**Thai**	[67]	1	1	1	1	1	1	1	1	1	NA	1	1	1	1	1,5	1	1	1	100,0%	Low
**Tobita**	[68]	1	1	1		1	0	0	1	NA	NA	1	1	1	1	1,5	0	0	0	60,0%	Moderate

**Toth**	[69]	1	1	1	1	0	0	0	1	NA	NA	1	1	1	1	1,5	0	0	1	65,7%	Moderate
**Twisk**	[70]	1	1	1		0	1	0	1	1	0	1	1	1	1	0	1	1	1	70,3%	Moderate
**Vrijlandt**	[71]	1	1	1	1	0	0	NA	1	NR	NR	1	1	1	1	0	0	1	1	59,5%	Moderate
**Wong**	[72]	1	1	1	1	0	0	0	1	NA	NA	1	1	1	1	0	0	1	0	57,1%	Moderate
**Yu**	[73]	1	1	1	1	NR	NR	NR	1	1	1	1	1	1	1	0	0	1	1	70,3%	Moderate
**Nagaya**	[74]	1	1	1	1	0	0	0	1	NA	NA	1	1	1	1	1,5	1	1	0	71,4%	Moderate
**López-Martínez**	[75]	1	1	1	1	0	0	0	1	1	NA	1	1	1	1	1,5	1	1	1	82,9%	Low
**Lefevre 2000**	[76]	1	1	1	1	0	1	NR	1	NR	0	1	1	1	1	1,5	1	1	1	78,4%	Low
**Carnethon**	[77]	1	0	1	1	1	1	1	1	1	NA	1	1	1	0	1,5	0	0	1	77,1%	Low
**Kim 2012**	[78]	1	1	1	1	0	0	0	1	NA	NA	1	1	1	1	1,5	0	0	1	65,7%	Moderate

[1]Ref. = Reference number
