# Supplementary material for: Correlates and Determinants of Cardiorespiratory Fitness in Adults: a Systematic Review
Source: Sports Med Open. 2019 Sep 3;5:39. doi: 10.1186/s40798-019-0211-2 (PMC6722171; doi:10.1186/s40798-019-0211-2)
Supplement: Supplementary file 2 — Sensitivity Analyses. (PDF 253 kb) [file 40798_2019_211_MOESM2_ESM.pdf]

Online Resource 2: Sensitivity Analyses

Correlates and determinants of cardiorespiratory fitness in adults: a systematic review

Johannes Zeiher*, Katherine J. Ombrellaro, Nita Perumal, Thomas Keil, Gert B. M. Mensink, Jonas D. Finger

* Corresponding author:

Johannes Zeiher
Unit of Health Behaviour
Department of Epidemiology and Health Monitoring
Robert Koch Institute
General-Pape-Straße 62-66
12101 Berlin, Germany
Email: zeiherj@rki.de

Tab OR2.1: Analyses of review results regarding the association of education and cardiorespiratory fitness according to sex

	Positive	Negative	Null	Total
Women	2	0	2	4
Men	3	0	2	5
Mixed	1	0	0	1
Total	6	0	4	10

Fisher's exact: p = 1.000

Tab OR2.2: Analyses of review results regarding the association of education and cardiorespiratory fitness (CRF) according to CRF measure

	Positive	Negative	Null	Total
Direct Vo2max	0	0	2	2
Indirect Vo2max	4	0	3	7
Other	0	0	1	1
Total	4	0	6	10

Fisher's exact: p = 0.467

Tab OR2.3: Analyses of review results regarding the association of education and cardiorespiratory fitness according to test machine

	Positive	Negative	Null	Total
Treadmill	2	0	3	5
Ergometer	2	0	3	5
Total	4	0	6	10

Fisher's exact: p = 1.000

Tab OR2.4: Analyses of review results regarding the association of age and cardiorespiratory fitness according to sex

	Positive	Negative	Null	Total
Women	0	13	2	15
Men	1	19	1	21
Mixed	1	6	0	7
Total	2	38	3	43

Fisher's exact: $p = 0.522$

Tab OR2.5 Analyses of review results regarding the association of age and cardiorespiratory fitness (CRF) according to CRF measure

	Positive	Negative	Null	Total
Direct Vo2max	1	26	0	27
Indirect Vo2max	0	6	1	7
Other	1	6	2	9
Total	2	38	3	43

Fisher's exact: $p = 0.055$

Tab OR2.6: Analyses of review results regarding the association of age and cardiorespiratory fitness according to test machine

	Positive	Negative	Null	Total
Treadmill	1	23	2	26
Ergometer	1	15	1	17
Total	2	38	3	43

Fisher's exact: $p = 1.000$

Tab OR2.7: Analyses of review results regarding the association of body mass index and cardiorespiratory fitness according to sex

	Positive	Negative	Null	Total
Women	0	9	1	10
Men	2	9	2	13
Mixed	1	0	0	1
Total	3	18	3	24

Fisher's exact: $p = 0.215$

Tab OR2.8: Analyses of review results regarding the association of body mass index and cardiorespiratory fitness (CRF) according to CRF measure

	Positive	Negative	Null	Total
Direct Vo2max	1	8	0	9
Indirect Vo2max	0	3	3	6
Other	2	5	0	7
Total	3	16	3	22

Fisher's exact: $p = 0.034$

Tab OR2.9: Analyses of review results regarding the association of body mass index and cardiorespiratory fitness according to test machine

	Positive	Negative	Null	Total
Treadmill	0	13	1	14
Ergometer	3	5	2	10
Total	3	16	3	24

Fisher's exact: $p = 0.091$

Tab OR2.10 Analyses of review results regarding the association of body mass index and cardiorespiratory fitness (CRF) according to body weight adjustment of CRF measure

	Positive	Negative	Null	Total
CRF adj. for weight	2	13	3	18
CRF not adj. for weight	1	5	0	6
Total	3	18	3	24

Fisher's exact: $p = 0.795$

Tab OR2.11: Analyses of review results regarding the association of physical activity index and cardiorespiratory fitness according to sex

	Positive	Negative	Null	Total
Women	5	0	2	7
Men	8	0	3	11
Mixed	1	0	0	1
Total	14	0	5	19

Fisher's exact: p = 1.000

Tab OR2.12 Analyses of review results regarding the association of physical activity index and cardiorespiratory fitness (CRF) according to CRF measure

	Positive	Negative	Null	Total
Direct Vo2max	5	0	5	10
Indirect Vo2max	0	0	0	0
Other	9	0	0	3
Total	14	0	5	19

Fisher's exact: p = 0.033

Tab OR2.13: Analyses of review results regarding the association of physical activity index and cardiorespiratory fitness according to test machine

	Positive	Negative	Null	Total
Treadmill	12	0	4	16
Ergometer	2	0	1	3
Total	14	0	5	19

Fisher's exact: p = 1.000

Tab OR2.14: Analyses of review results regarding the association of smoking and cardiorespiratory fitness according to sex

	Positive	Negative	Null	Total
Women	1	4	3	8
Men	0	8	4	12
Mixed	0	1	1	2
Total	1	13	8	22

Fisher's exact: $p = 0.762$

Tab OR2.15 Analyses of review results regarding the association of smoking and cardiorespiratory fitness (CRF) according to CRF measure

	Positive	Negative	Null	Total
Direct Vo2max	0	5	3	8
Indirect Vo2max	1	0	3	4
Other	0	8	2	10
Total	1	13	8	22

Fisher's exact: $p = 0.038$

Tab OR2.16: Analyses of review results regarding the association of smoking and cardiorespiratory fitness according to test machine

	Positive	Negative	Null	Total
Treadmill	1	9	6	16
Ergometer	0	4	2	6
Total	1	13	8	22

Fisher's exact: $p = 1.000$

Tab OR2.17: Analyses of review results regarding the association of waist circumference and cardiorespiratory fitness according to sex

	Positive	Negative	Null	Total
Women	0	4	1	5
Men	0	5	0	5
Mixed	0	0	0	0
Total	0	9	1	10

Fisher's exact: p = 0.500

Tab OR2.18 Analyses of review results regarding the association of waist circumference and cardiorespiratory fitness (CRF) according to CRF measure

	Positive	Negative	Null	Total
Direct Vo2max	0	4	0	4
Indirect Vo2max	0	3	1	4
Other	0	0	0	0
Total	0	7	1	8

Fisher's exact: p = NA < 10 samples. Two samples did not report CRF measurement.

Tab OR2.19: Analyses of review results regarding the association of waist circumference and cardiorespiratory fitness according to test machine

	Positive	Negative	Null	Total
Treadmill	0	5	1	6
Ergometer	0	4	0	4
Total	0	9	1	10

Fisher's exact: p = 0.400
