# Supplementary material for: Correlates and Determinants of Cardiorespiratory Fitness in Adults: a Systematic Review
Source: Sports Med Open. 2019 Sep 3;5:39. doi: 10.1186/s40798-019-0211-2 (PMC6722171; doi:10.1186/s40798-019-0211-2)
Supplement: Supplementary file 3 — Limited Search Update. (PDF 210 kb) [file 40798_2019_211_MOESM3_ESM.pdf]

Online Resource 3: Limited Search Update

Correlates and determinants of cardiorespiratory fitness in adults: a systematic review

Johannes Zeiher*, Katherine J. Ombrellaro, Nita Perumal, Thomas Keil, Gert B. M. Mensink, Jonas D. Finger

*** Corresponding author:**
Johannes Zeiher
Unit of Health Behaviour
Department of Epidemiology and Health Monitoring
Robert Koch Institute
General-Pape-Straße 62-66
12101 Berlin, Germany
Email: zeiherj@rki.de
Phone: 0049 30 18754 3775

Table OR3.1 Results summary: search update

Author (Date) [Reference]	Country	Study design	Sampl e-size	% female	Age range/ mean age (in years)	CRF test	Results	
Chen et al. (2017) [159]	US	cross-sectional	3,292	48%	40-49	submaximal treadmill test	**Men**[1]<br>Ethnicity: **+/0/-**[2]<br>Diabetes: **-** (obese)<br>Homocysteine: **-** (normal weight, overweight)<br>Total cholesterol: **0** (normal weight) / **-** (overweight)<br>Vitamin B12: **-** (obese)<br>Smoking: **+** (overweight)	**Women**[1]<br>Ethnicity: **+/0/-** [3]<br>Diabetes: **+** (normal) / **0** (overweight)<br>Homocysteine: **-** (normal weight)<br>Folate: **+** (obese)<br>C-reactive protein: **-** (obese)<br>Total cholesterol: **-** (overweight)<br>Hemoglobin: **+** (overweight) / **0** (obese)<br>Vitamin B12: **-** (obese)<br>Smoking: **0** (overweight)<br>Alcohol intake **+** (overweight)
Jackowski et al. (2017) [160]	Canada	longitudinal	61	38%	~47[4]	maximal treadmill test	Biological age: **0**<br>Biological age[2]: **0**<br>Height : **+**<br>Sex: **0**<br>Cardiometabolic risk: **0**	
Knaeps et al. (2018) [161]	Nether-lands	longitudinal	399	36%	56	maximal cycle ergometer test	cardiometabolic risk score: **-**<br>cardiometabolic risk score (no adiposity): **-**<br>Waist circumference: **-**<br>Fasting glucose: **-**<br>HDL cholesterol: **+**<br>Triglycerides : **-**<br>Diastolic blood pressure: **-**<br>Systolic blood pressure: **-**	
Shouval et al. (2017) [162]	Israel	longitudinal	16,334	30%	46	maximal exercise treadmill test	**Men**<br>Anemia (WHO criteria): **0**<br>Age: **-**<br>Body mass index: **-**<br>Lack of physical activity: **-**<br>Hypertension: **-**<br>Estimated glomerular filtration rate: **-**<br>Total cholesterol: **-**	**Women**<br>Anemia (WHO criteria): **-**<br>Age: **-**<br>Body mass index: **-**<br>Lack of physical activity: **-**<br>Hypertension: **-**<br>Estimated glomerular filtration rate: **0**<br>Total cholesterol: **-**

Tripette et al. (2018) [163]	Japan	cross-sectional	867	86%	23-88	maximal ergometer test	**Men**<br>Caffeine consumption: **0**	**Women**<br>Caffeine consumption: **+**
Tsou et al. (2018) [164]	US	cross-sectional	449	45%	20-50	submaximal treadmill test	**Men**<br>Arachidic Acid: **-**<br>Docosanoic Acid: **-**<br>Arachidonic Acid: **-**<br>Docosatetraenoic Acid: **-**<br>Age: **-**<br>Smoking (Y/N): **0**<br>Diastolic blood pressure (mmHg): **-**<br>Body mass index: **0**<br>C-reactive protein: **0**<br>Homeostatic Model Assessment - Insulin Resistance: **0**	**Women**<br>Arachidic Acid: **0**<br>Docosanoic Acid: **0**<br>Arachidonic Acid: **-**<br>Docosatetraenoic Acid: **0**<br>Age: **0**<br>Smoking (Y/N): **0**<br>Diastolic blood pressure (mmHg): **0**<br>Body mass index:**0**<br>C-reactive protein:**0**<br>Homeostatic Model Assessment - Insulin Resistance:**0**
van der Velde et al. (2017) [165]	Nether-lands	cross-sectional	2,024	51%	40-75	submaximal cycle ergometer test	**Men**<br>Sedentary time: **-**<br>Sedentary Breaks (10/d): **0**<br>Average Sedentary Bout Duration (min): **0**<br>Prolonged Sedentary Bouts (n/d): **0**	**Women**<br>Sedentary time: **-**<br>Sedentary Breaks (10/d): **0**<br>Average Sedentary Bout Duration (min): **0**<br>Prolonged Sedentary Bouts (n/d): **0**

+: positive association; −: negative association; 0: null association

[1]In this study results were reported stratified for weight status (normal weight, overweight, obese) and potential correlates were only analysed in the multivariable model, if they showed a significant bivariate association (term in parenthesis indicates the strata).

[2]Negative association (Reference: ’non-Hispanic white’) of CRF with ‘other Hispanic’ and ‘other race’, no association with, ‘non-Hispanic black’ and ‘Mexican american’ among normal weight participants; no association with, ‘non-Hispanic black’, ‘Mexican American’, ‘other Hispanic’ and ‘other race’ among overweight and obese participants.

[3]Negative association (Reference: ’non-Hispanic white’) of CRF with ‘non-Hispanic black’, no association with ‘Mexican American’, ‘other Hispanic’ and ‘other race’ among normal weight participants; no association with, ‘non-Hispanic black’, ‘Mexican American’, ‘other Hispanic’ and ‘other race’ among overweight participants; negative association of CRF with ‘non-Hispanic black’ and ‘other race’, no association with ‘Mexican american’ and ‘other Hispanic’ among obese participants.

[4]Mean age for low CRF group (n=26): 48.62; mean age for high CRF group (n=35): 45.99.
